# Biomechanical costs of reaching movements bias perceptual decisions

**DOI:** 10.1186/1471-2202-14-S1-P408

**Published:** 2013-07-08

**Authors:** Encarni Marcos, Ignasi Cos, Paul Cisek, Benoît Girard, Paul FMJ Verschure

**Affiliations:** 1 Laboratory of Synthetic Perceptive, Emotive and Cognitive Systems (SPECS), Universitat Pompeu Fabra, Barcelona, 08018, Spain; 2 Institut de Systèmes Intelligents et de Robotique (ISIR), Université Pierre et Marie Curie, Paris, 75005, France; 3 UMR-7222, Centre National de la Recherche Scientifique (CNRS), Paris, 75005, France; 4 Department of Physiology, Université de Montréal, Montreal, Quebec, Canada, H3T 1J4; 5 Institució Catalana de Recerca i Estudis Avançats (ICREA), Barcelona, 08010, Spain

## 

Perceptual decision-making has been widely studied using tasks in which subjects are presented with a visual stimulus and are instructed to select between two alternative options, based on a visual feature of the stimulus. Several studies have reported that the subjects' performance depends on the difficulty to indentify that specific feature. However, if we view decision-making as a continuous process that includes not only perceptual discrimination but also motor action one would expect that the cost of this action will have an impact on decision-making during the action preparation phase. We investigated this hypothesis with a combined theoretical and experimental approach.

We setup a psychophysical task with human subjects, consisting of two blocks. In the first block, subjects either had to perform one of four right- or left-ward reaching movements, each with different biomechanical costs, or to make decisions between these right or left reaching movements. The movements were organised in two arrangements: one in which the movement towards the right implied a larger biomechanical cost than the movement towards the left, and one in which the costs were reversed. In the second block, subjects performed a moving dots perceptual discrimination task with different levels of coherence (right or left motion), and reported their decision with a reaching movement to the right or to the left using one of the aforementioned manipulations. As previously reported [1], we observed that when subjects freely chose between reaching movements of varying biomechanical cost, they exhibited a strong bias towards those movements with lower cost. Here, we report that this bias also influenced the motion-discrimination task, as subjects exhibited a tendency to perform the less costly movement when the ambiguity of the stimulus was high. The influence of the biomechanical bias declined as the ambiguity decreased. These results suggest that biomechanical costs are assessed and influence decisions even when the correct response does not depend on the properties of the motor apparatus.

To study how visual and biomechanical information might be integrated to make decisions we use a mean-field approach of a binary decision-making model [2].The model consists of two populations of excitatory neurons that are responsive to left or right motion and that compete through mutual inhibition. We propose that even if the decision itself should exclusively depend on a single factor (motion direction), additional factors, such as biomechanics, may also influence the decision-making process. Simulation results show that a constant signal related to the biomechanical cost is necessary and sufficient to explain the probability of choice and reaction time in our motion discrimination task. Interestingly, the model shows that for same motion coherence the activation of the network is higher when the dots move toward the direction reported with the less costly movement than when they move towards the more costly one. An open question is whether the neural activity in the supplementary motor area, dorsal premotor cortex and primary motor cortex exhibit correlates of this biomechanical factor.
